# Commentary: Unsettling friendship and using friendship to unsettle

**DOI:** 10.1177/0042098017730543

**Published:** 2017-09-26

**Authors:** Halleh Ghorashi

**Affiliations:** Vrije Universiteit Amsterdam, The Netherlands

**Keywords:** imagining change, othering discourse, unsettling normalised dichotomies, ‘unusual friendships’, urban relationalities

## Abstract

Since the early 2000s there has been an undeniable global escalation of negative othering discourses concerning migrants and refugees. The fixation on ethnic difference in these discourses blinds us toward possible sources of connection. To unsettle this essentialist discourse of othering, we need to consider practices that denormalise the taken-for-granted taxonomies of the Self and the Other at their cores and rethink conditions for connection. Urban relational initiatives, experiences and narrations could provide interesting perspectives for exploring new possibilities for connection in liquid modern times, where old-fashioned collective categories lost their function. A multilayered, non-centric, non-celebratory approach of friendship as an empirical and conceptual frame provides a refreshing angle for capturing the multiplicity of everyday urban interactions. The contributions to this special issue provide insights toward enlarging our imaginings of the myriad ways that friendship as a concept and an empirical reality is enabling and constraining relationality in diverse urban settings. Here, I also argue for the importance of ‘unusual’ friendships and their potential to unsettle normalised practices of othering, thereby producing new narratives of connections in a variety of urban settings. All these small yet significant acts of friendship might be either ‘chained’ strategically to promote a collective alternative to normalised practices or ‘chained’ in an invisible manner, serving as existing subtle and modest struggles in imagining social change.

## 摘要

自本世纪初以来，与移民和难民相关的消极他者化话语在全球范围内无疑加剧了。这些话语聚焦在族群差异上，使我们看不到可能的关联源。要消解这种本质主义的他者化话语，我们需要考虑那些从核心消解自我与他者两分的做法，并重新思考关联条件。在一切皆流动的现代，老式的集合类别失去了其功能，城市关系举措、经验和叙事可为探索新的关联可能性提供有趣的视角。从多层化、无中心、非庆贺的方式看待友谊，将它作为一种经验的、概念性的框架，能为捕捉城市日常互动的多重性提供一个新鲜的视角。友谊作为一个概念和一种经验现实，在多样的城市场景中以各种各样的方式促成又限制了人际关系；本专辑收录的文章为扩展我们对这些方式的想象提供了洞见。本人在这里也认为“非寻常”友谊很重要，并有潜力消解常态的他者化行为，从而在各种城市场景中产生新的关联叙事。所有这些虽然微小但又意义重大的友谊行为可能以战略性的方式“链接”，从而促进常态化行为之外的集体行为，也可能以不可见的方式“链接”，从而成为社会变革想象过程中已有的微妙斗争。

Bauman (2000) argues that the era of liquid modernity is marked by the mobility of ideas, people and resources as well as an increasing sense of insecurity and growing structures of inequality (providing more space for mobility to some over others). At the same time we observe the increasing mobility of people across nation-state borders. Steven Vertovec (2007) defines the current condition of Western states as one of ‘super-diversity’: a state that is less ordered and less tangible than its late 20th-century counterpart. Global events in recent decades, such as 11 September 2001, have brought the intersection of insecurity and super-diversity into a completely different light, leading to increasingly visible negative views on migrants and efforts to limit their presence in most Western societies. Since the early 2000s there has been an undeniable escalation of negative othering discourses concerning the Muslim diaspora. And since 2015 refugees have moved to the centre of public space in many Western societies, where they are dominantly portrayed as ‘space invaders’. The othering discourse on migrants is not new, but there are two significant differences in this time compared with the decades preceding the turn of the century. First is the blatant manner in which negative sentiments toward migrants of colour as ethnic or racialised Others prevails in the public and political space of many Western countries. If the dominant discourses in previous decades were ones of tolerance of one form or another, the tone of current public debates is openly aggressive. Second, despite the visibility of various othering practices in Europe and beyond, the immense power of these practices lies in their normalising capacity, their invisibility, in how they become part of the taken-for-granted practices of everyday life. This invisible normalising working of power is one of the central features of late modern societies (Bauman, 2000).

Despite these differences with previous decades, Prasad and Prasad argue for the historical embeddedness of these discourses, which can be traced back to the colonial legacy. This legacy informs ‘the social and cultural construction of a fundamental *ontological* distinction between “the west” and “the non-west”’ (2002: 61). Within the constructed binaries of difference, the ethnic Other has been considered not only absolutely different but also inferior to the norm of the ethnic Western Self. In the same vein, Anthias (2013: 2) argues there is a strong ‘culturalization of social relations’ that leads to the reification of difference as dangerous and blinds us to other broader sources of exclusion. This fixation on ethnic difference also blinds us toward possible sources of connection. To unsettle this ‘culturalist discourse of othering’, we need to consider practices that de-normalise the taken-for-granted taxonomies of the Self and the Other at their cores and rethink conditions for connection.

## Rethinking the sources of societal connection

A growing body of literature criticises the essentialising, culturalist and sedentarist approaches to ethnicity and migrant communities (Ghorashi, 2016). These studies focus on the relational and multiple aspects of connections in various locations. Urban settings such as cities and neighbourhoods are increasingly considered locations that enable us to experiment with the relational capacity of connectedness in its multilayeredness. Yet metropolitan cities also often become idealised as havens of mobility, hybridity and breathing ground for creative industries. These approaches often conceal the contradictory existence of centric and marginal spaces in the cities in addition to the simultaneous presence of inclusionary and exclusionary discourses and practices. Favell (2008), for example, shows that Amsterdam has been less permissive and open than it presents itself. In an ethnographic comparison between different European cities, Favell argues that Amsterdam is a deeply regulated and controlled city in which some processes of social and ethnic closure are ingrained in daily interactions. Thus, in the context of growing mobility and diversity, there is a need for studies that capture the complexity and layered presence of contradictory processes in cities and that go beyond the privileged-centric approaches of diversity, cohesion and creativity. A multi-layered, non-centric, non-celebratory approach of friendship as an empirical and conceptual frame provides a refreshing angle to capture the multiplicity of everyday urban interactions.

This special issues’ diverse and substantive descriptions of practices and processes of (dis)connectedness in urban locations is a very welcome contribution to the existing knowledge. The specific critical approach of friendship found within is insightful and rich, and it furthers our thinking in various ways. The articles all deconstruct a one-dimensional approach of friendship ingrained in governmental policies toward social cohesion or integration. Most are critical of the overmanagement of intense engagement as the recipe for social cohesion. However, their routes in this deconstruction differ, adding to the richness of the special issue. The authors engage with the concept of friendship to unsettle a variety of binaries that are often taken for granted in academic works and policy making. By doing so they show the complexity and the multilayeredness of interpersonal relationships in super-diverse urban contexts. The common thread running throughout is the importance of capturing the richness of interpersonal everyday togetherness in a variety of urban environments. Attention is given to the banality of intercultural engagement involving the simultaneous presence of familiarity and distance. Furthermore, this special issue provides not only a critique of but also insightful alternatives to the frame of social cohesion as the dominant frame in managing diversity.

Most contributions in this special issue challenge the normalised notion of friendship as exclusively positive. Some focus on conditions of marginality and precariousness, showing the difficulty and even danger of local intimacy. Loren B Landau (2018, this issue) for example refers to ‘communities of convenience’ as spaces in which local intimacy is neither possible nor desirable. Landau’s article show the importance of social connections as the source of support and survival and explores the possibilities of flexible and intentionally fleeting friendship as ‘tactical cosmopolitanism’. Instead of belonging to multiple communities, this kind of unbounded connectedness enables people to be in a place but not of it. Similar discussion is present in Olivia Killias’ (2018, this issue) article about Iranian student residents of a Malaysian multiethnic apartment building. These students’ specific in-between positionality as free in Malaysia but not free from Iranian political influence creates a precarious situation that makes close social ties with other Iranians risky. Anita Harris’ (2018, this issue) contribution shows how young people also create ways of togetherness beyond and beneath the imperatives of social cohesion initiatives. Somewhat comparable with the unbounded connectedness presented in Landau’s article, Harris refers to a kind of ‘light touch sociality’ through which intimate mixing coexists with prejudiced attitudes and practices. She thus provides insights into ‘unpanicked multiculturalism’ in which support and engagement go hand in hand with friendly distance or ‘companionable silence’. Similarly, Michele Lobo (2018, this issue) explores the co-presence of ‘quiet politics’ and affective engagement in Northern Australia. By favouring non-Western and marginalised geographies of friendship, Lobo challenges ‘metrocentricity’ or glorification of creative clusters in the city centres. She shows how engaged art projects at the margins of these centres provide insights to friendships in terms of ‘affective intensities’ with a ‘light touch’ rather than strong emotional bonds.

Some articles focus on the simultaneous presence of competing and conflicting experiences in urban micro-interactions. Megha Amrith (2018, this issue) shows how urban relationships for low-income migrants include a coexistence of companionship and emotional solidarity, which are essential for migrants’ survival but are also ingrained with conflict and competition. She argues for different moralities of friendship in which coexistence of contradictory experiences and feelings are practiced in diverse shared spaces. Shanti Robertson (2018, this issue) unsettles simplistic dichotomies of conceptualisations such as co-ethnic ‘bonding’ and cross-ethnic ‘bridging’ and argues for ‘translocal subjectivities’, shaped through culturally and historically situated and overlapping forms of friendships that are ‘both multiply located and transformed through transnational mobility’. Darya Malyutina (2018, this issue) also criticises overemphasising ethnicity as a marker of difference by showing that friendship ties can be a source of migrant support and empowerment against marginality, but those ties also reflect power relations and reproductions of exclusionary practices.

By analysing retirees’ collective activities in Beijing public parks, Lisa Richaud (2018, this issue) convincingly challenges two conceptual poles – ‘face’ (i.e. communitarian) and ‘faceless’ (i.e. anonymous) – in the context of urban interactions and experiences. By unpacking the experiences of elderly Chinese people who carry memories of political violence from the Cultural Revolution, Richaud calls for consideration of the simultaneous presence of multiplicity in sharing a specific public space and pleasure in a collective activity while maintaining anonymity or partial engagement (or even silence). Richaud’s article brought me back to almost two decades ago, when I encountered elderly men in China playing games together in absolute silence. I was mesmerised by the combination of play and silence, feeling the presence of heavy memories of the past. The focus on silence in several articles of this special issue (Richaud, 2018, but also Harris’s ‘companionable silence’ (2018)) is quite insightful in an academic space that privileges language and conversations for studying engagement and connectedness. Space and consideration for silence is especially important for marginalised groups who do not have the privilege of occupying the centre with their words, or for refugees with traumas (Ghorashi, 2008). The same could be said for people who are in vulnerable situations because of their health such as people with dementia, as presented by Phillips and Evans (2018, this issue). Through analysing a city initiative to support people with dementia, the authors introduce curiosity as the catalyst for bringing people together and encouraging them to ‘take notice’ and ‘connect’. For curiosity to work as a catalyst for friendship, the authors borrow Sennett’s (2012) means of ‘listening well’ and ‘behaving tactfully’. They conclude by emphasising that, although relationships have become more ‘fleeting and transient’, this does not mean friendships are not good or lasting.

## Unusual friendships

Weaving insights from the articles in this special issue is helpful in rethinking practices and engagements for tackling the dominance of normalised culturalist discourses of othering through reimagining friendship and democracy. One foundation for rethinking sources of connection is that of revitalising our notion of democracy, or in Gidden’s (1999) words: ‘democratizing democracy’. A large body of literature discusses the illusions, deficits and limitations of representational democracy (e.g. Baudrillard, 2009; Žižek, 2011). Yet a deeper notion of participatory democracy provides some points of inspiration. To unsettle the normalised practices of inequality and exclusion, Iris M Young (2002) suggests a deeper notion of democracy that includes situated inclusive conversations in which personal, political and contextual aspects of interaction meet. Inclusion means being sensitive to ‘unusual voices’ and ‘listening to silences’ (Medina, 2013). The obvious first step would be to focus on the ‘commitment to preserving the allegiance of citizens, including electoral minorities, *despite* majority rule’ (Allen, 2004: xix). This requires a politics of engagement (besides a politics of justice) that is not obligatory but is based on public feelings of connectedness and curiosity (Amin, 2012). Friendship has been considered important for public engagement inspired by Aristotle’s fantasy of timocracy, or a city of brothers (Allen, 2004). In the era of liquid modernity, the traditional ties of connections (to a given community) are being displaced by differentiated sources of affective amplifications, requiring a suspension of judgement regarding the level of authenticity of those affections (Amin, 2012). But how to imagine friendship and connectedness when the Other is considered as a ‘potential enemy’, living inside the nation but not entirely belonging to it?

In current European societies where the Other is categorised as ‘unusual’, ‘strange’, ‘untrustworthy’, ‘unwanted’ or ‘abnormal’, friendship could become a powerful means to unsettling the dichotomous and normalised practice of othering. Below, a personal anecdote shows a strategic uses of friendship that explicitly unsettles dichotomies of difference considered ‘odd’, such as being friends with the assumed ‘enemy’.

Being a regular visitor at the gym creates connections through presence, even without much talk. Once I was in the sauna with a friend when a guy (also a regular) asked us, ‘May I ask you a personal question? Are you twins?’ We both had to laugh – we often hear that we look alike, but no one had ever asked if we were twins before. Then I said, ‘We’re not twins. I’m an Iranian Muslim and my friend is an American Jew’. A profound silence fell, probably a sign of great shock. The guy did not talk the rest of the time in the sauna and seemed to have gone into deep thought.

Presenting a close friendship with ‘a potential enemy’ in a rather exaggerated manner by keeping the nuances out of the presentation (I was born a Muslim yet am not practicing, and my friend is a Jew who is married to an Iranian man) is a way to de-normalise the essentialist foundation of assumptions of difference that are taken for granted. In this way an ‘unusual friendship’ is used as a strategy to stimulate the imagining of connections beyond expected assumptions. Using examples such as this in various urban physical sites of interaction, but also in cyberspace, is another way of presenting a story of ‘a strange connection’ to shake the fixed taxonomies of difference. This strategy is inspired by Judith Butler’s (2008) discussion of *drag*. Butler argues that gender categories are reified through repetition. ‘This repetition is at once a re-enactment and re-experiencing of a set of meanings already socially established; and it is the mundane and ritualised form of their legitimation’ (Butler, 2008: 191). For Butler, *drag* unsettles this normalised and fixed presented reality of gender norms. *Drag* thus resists the violence performed by the normalised gender ‘reality’ of difference (Butler, 2008). In the same vein, I argue that the ‘unusual’ or ‘odd’ examples of friendship introduced above and discussed in this special issue have the same capacity to break the persistence of cultural boundaries of difference through repetition. However, it is worthwhile to explore further the capacity of the strategic use of friendship in its potential of unsettling normalised practices of othering to produce new narratives of connections in variety of urban settings.

When the power of exclusion works through repetition and is manifested in the daily normalisation of our actions, we need to consider agency in terms of small actions, or micropolitics, taken by individuals and groups in their daily interactions. By repeatedly presenting ‘unusual stories of friendship’ and practicing ‘unconventional public engagements’ in different urban locations and spaces, the subtle and taken-for-granted power of the normalisation of othering can be subverted. In this way agency is manifested in partial and temporal movements that break away from the subtle workings of power rather than in a grand movement against oppressive power that leads to a utopic society (Zanoni and Janssens, 2007). Repetition of small unsettling choices and demonstrations comes close to what Judith Butler suggested as a ‘strategy of subversive repetition’. This, what I call *unsettling politics of connection* through unconventional engagement, is a powerful way to subvert the subtle and ungraspable power of normalisation (see also Ghorashi, 2014). Although we need to have more than individual responses (i.e. collective responses) for challenging societal issues, as Young (2006) argues, this does not mean that we have to concentrate on collective actions in the traditional way. Medina (2013) proposes ‘chained actions’ as alternatives for collective actions and argues that all individuals ‘have (typically plenty of) particular things to do in the work toward justice’ or towards a more connected society. These actions might either be ‘chained’ strategically to promote a collective alternative to normalised practices or ‘chained’ in an invisible manner, serving as existing subtle and modest struggles in imagining social change. ‘These “small, fragmented, and sometimes contradictory efforts by people to change their lives” may be small, and at times short-lived, but they are significant’ (Krause 1983: 54, in Stall, 2010: 546). The contributions of this special issue provide insights particularly in enlarging our imaginary of the variety of ways that friendship as a concept and an empirical reality is enabling and constraining relationality in diverse urban settings.
